# Supplementary material for: The Systems Biology Research Tool: evolvable open-source software
Source: BMC Syst Biol. 2008 Jun 29;2:55. doi: 10.1186/1752-0509-2-55 (PMC2446383; doi:10.1186/1752-0509-2-55)
Supplement: Additional file 1 — SBRT Archive. An archive of the current version of the Systems Biology Research Tool. [file 1752-0509-2-55-S1.zip › sbrt-1.4.0/doc/developers_guide/api/sbrt/shell/text/PipeListFormat.html]

PipeListFormat


|  |  |  |
| --- | --- | --- |
| **Overview** \| **Package** \| **Class** \| **Use** \| **Tree** \| **Deprecated** \| **Index** \| **Help** | |  |
| **PREV CLASS**   **NEXT CLASS** | **FRAMES**    **NO FRAMES**     **All Classes** |  |
| SUMMARY: NESTED \| FIELD \| CONSTR \| METHOD | DETAIL: FIELD \| CONSTR \| METHOD |  |


---


## sbrt.shell.text Class PipeListFormat<E>

```
java.lang.Object
  sbrt.shell.text.AbstractListFormat<E>
      sbrt.shell.text.PipeListFormat<E>
```

**Type Parameters:**: `E` - the type of element.

**All Implemented Interfaces:**: Formatter<java.util.List<? extends E>>, ListFormat<E>, Parser<java.util.List<E>>

---

``` public final class PipeListFormat<E> extends AbstractListFormat<E> ```

This class is used to define a format for pipe-delimited
lists. If a list is composed of elements
*a, b, c*,
its string representation would be
"`a | b | c`".

**Author:**
:   This class was written and documented by
    Jeremiah Wright while in the Wagner lab.

---

**Constructor Summary**

`PipeListFormat(Formatter<E> elementFormatter,
Parser<E> elementParser)`
  
          Constructs a new pipe-delimited list format with the
provided element format.

`PipeListFormat(SimpleFormat<E> elementFormat)`
  
          Constructs a new pipe-delimited list format with the
provided element format.


| **Method Summary** | |
| --- | --- |
| `static PipeListFormat<java.lang.String>` | `getBasic()`             Returns a `PipeListFormat` that does not format or parse the elements supplied to it. |

**Methods inherited from class sbrt.shell.text.AbstractListFormat**

`format, getDelimiter, getElementFormatter, getElementParser, getRegex, parse`

**Methods inherited from class java.lang.Object**

`clone, equals, finalize, getClass, hashCode, notify, notifyAll, toString, wait, wait, wait`

**Methods inherited from interface sbrt.shell.text.Formatter**

`format`

**Constructor Detail**

### PipeListFormat

```
public PipeListFormat(SimpleFormat<E> elementFormat)
```

:   Constructs a new pipe-delimited list format with the
    provided element format.

    **Parameters:**: `elementFormat` - the format of the list-elements.

---


### PipeListFormat

```
public PipeListFormat(Formatter<E> elementFormatter,
                      Parser<E> elementParser)
```

:   Constructs a new pipe-delimited list format with the
    provided element format.

    **Parameters:**: `elementFormatter` - the formatter for the list-elements.: `elementParser` - the parser for the list-elements.


**Method Detail**

### getBasic

```
public static final PipeListFormat<java.lang.String> getBasic()
```

:   Returns a `PipeListFormat` that does not
    format or parse the elements supplied to it. In other
    words, it returns a `PipeListFormat` with
    the most basic possible functionality. Empty strings are not
    allowed however.

    :   **Returns:**: an `PipeListFormat` that does not format or parse the elements supplied to it.


---


|  |  |  |
| --- | --- | --- |
| **Overview** \| **Package** \| **Class** \| **Use** \| **Tree** \| **Deprecated** \| **Index** \| **Help** | |  |
| **PREV CLASS**   **NEXT CLASS** | **FRAMES**    **NO FRAMES**     **All Classes** |  |
| SUMMARY: NESTED \| FIELD \| CONSTR \| METHOD | DETAIL: FIELD \| CONSTR \| METHOD |  |


---
